# Effects of partial substitution of chemical fertilizer with organic manure on the activity of enzyme and soil bacterial communities in the mountain red soil

**DOI:** 10.3389/fmicb.2023.1234904

**Published:** 2023-09-05

**Authors:** Zerang Lu, Yun Zhou, Yongmei Li, Chunpei Li, Mei Lu, Xuemei Sun, Zhizhang Luo, Jixia Zhao, Maopan Fan

**Affiliations:** ^1^College of Resources and Environment, Yunnan Agricultural University, Kunming, China; ^2^College of Ecology and Environment, Yunnan University, Kunming, China

**Keywords:** fertilizer, maize, soil properties, soil bacterial community, soil enzyme activity, mountain red soil

## Abstract

**Introduction:**

The partial substitution of chemical fertilizer with organic manure takes on a critical significance to enhancing soil quality and boosting sustainable agricultural development. However, rare research has studied the effects of partial substitution of chemical fertilizer with organic manure on soil bacterial community diversity and enzyme activity in maize field in the mountain red soil region of Yunnan.

**Methods:**

In this study, four treatments were set up in which chemical fertilizer (the application rates of N, P_2_O_5_ and K_2_O were 240, 75 and 75 kg·ha^−1^, respectively) was substituted by 10% (M10), 20% (M20), 30% (M30) and 40% (M40) of organic manure with equal nitrogen, as well as two control treatments of single application of chemical fertilizer (M0) and no fertilization (CK). The maize (*Zea mays* L.) crop was sown as a test crop in May 2018. The effects of partial substitution of chemical fertilizer with organic manure on soil physicochemical properties, soil bacterial community diversity and enzyme activity were studied.

**Results:**

The activities of Cellulase (CBH), Invertase (INV) and β-glucosidase (BG) increased with the increase of organic manure substitution ratio. The activities of β-1,4-N-acetylglucosaminidase (NAG), Urease (URE), and leucine aminopeptidase (LAP) also had the same trend, but the highest activities were 159.92 mg·g^−1^·h^−1^, 66.82 mg·g^−1^·h^−1^ and 143.90 mg·g^−1^·h^−1^ at 30% substitution ratio. Compared with CK and M0 treatments, Shannon index increased notably by 82.91%–116.74% and 92.42%–128.01%, respectively, at the organic manure substitution ratio ranging from 10% to 40%. Chao1 and ACE index increased significantly at the organic manure substitution ratio ranging from 10% to 30%. *Proteobacteria* was the dominant phylum in all treatments, the relative abundance of *Proteobacteria* decreased as the organic manure substitution ratio increased. Redundancy analysis showed that microbial biomass C was the main factor affecting the bacterial community composition under partial replacement of chemical fertilizer treatment, while *Actinobacteria* was the main factor affecting the enzyme activity. In addition, the maize yield of M30 and M40 treatments was significantly higher than that of CK and M0-M20 treatments, and the yield of M30 treatment was the highest, reaching 7652.89 kg·ha^−1^.

**Conclusion:**

Therefore, the partial substitution of chemical fertilizer with organic manure can improve soil biological characteristics, while increasing bacterial community diversity and soil enzyme activity. Therefore, a thirty percent organic manure substitution was determined as the optimal substitution ratio for maize farmland in the mountain red soil area of Yunnan, China.

## 1. Introduction

Fertilizers are the indispensable agricultural measure to improve soil fertility and sustaining crop yield (Xiang et al., 2020), High levels of agricultural production are heavily determined by fertilizers (Muhammad et al., 2020). However, excessive application of chemical fertilizer is a common and serious problem in China, and overuse of chemical fertilizers exceeds crop demand (Zhu et al., 2019), causing soil degradation (e.g., soil acidification, soil structure deterioration (Guo et al., 2010), reducing soil organic carbon (SOC) content and changing the carbon (C) pool (Li et al., 2014), soil pollution and drastic reduction of soil biodiversity and other environmental problems (Wu et al., 2020)). The above soil environmental problems jeopardize entire agroecosystems, reducing the sustainability of crop production (Kim et al., 2022). Thus, how to ensure high and stable yield while controlling the application of chemical fertilizers is of critical significance to achieving sustainable agricultural development.

Manure has been confirmed as a viable alternative to chemical fertilizers (Du et al., 2020), which may contribute to soil physical, chemical and biological characteristics. It is capable of improving soil quality and structure, stimulating soil enzyme activity, and enhancing functional diversity and richness of soil community structure (Zhao et al., 2016; Hou et al., 2022a,b). However, the fertilizer efficiency of manure is generally low and difficult to control; farmers are inclined to use chemical fertilizers instead of manure to maintain crop yields in agricultural production. Accordingly, the combined application of manure (organic manure) and chemical fertilizer has been proven as a sustainable fertilizer management strategy (Zhao et al., 2014). The partial substitution of chemical fertilizer with organic manure is effective in increasing the efficiency of crop resource use and enhancing the quality of agricultural products. On that basis, the input of chemical fertilizers can be reduced, while soil quality and sustainable agricultural production can be maintained (Gattinger et al., 2012; Ji et al., 2021). Existing research has suggested that partial replacement of chemical fertilizers with organic fertilizers can expedite microbial growth while changing soil microbial community structure and diversity (Qiao et al., 2019; Xiang et al., 2020), thus increasing enzyme activity (Lazcano et al., 2012) and soil fertility and crop productivity in the long term. The optimal substitution ratio remains unclear, especially for the optimal substitution ratio of organic manure in maize farmland in the mountain red soil area of Yunnan Province, though the advantages of organic manure have been generally recognized. Previous research has indicated that a higher substitution ratio leads to higher rice yields in southern China (Bi et al., 2009), and Huang et al. (2010) reported that 50% organic manure instead of fertilizer treatment yields higher maize than single fertilizer, or 100% organic fertilization in the North China Plain. Thus, a wide variety of crops have different responses to the combined application ratio of organic fertilizer and chemical fertilizer, and the effect of some organic manure instead of chemical fertilizer on soil microbes and crop yield should be studied in depth.

Changes in soil microbial community abundance and composition can effectively indicate soil biochemical processes and crop productivity in agricultural systems (Liu et al., 2018), and soil microbes are critical to enhancing soil physicochemical properties, controlling soil microbial community and diversity, and maintaining soil quality and fertility (Tang et al., 2020). Appropriate community structure, rich diversity, and high microbial activity have been confirmed as crucial factors for maintaining soil ecosystem and productivity (Zhao et al., 2014). Existing studies reported that the microbial biomass and diversity of manure and compost soil more obvioulsy increase than mineral fertilizer, but a wide variety of fertilization methods and organic manure types have different responses to soil microbial biomass and diversity (Geisseler et al., 2017; Wang et al., 2018). Moreover, soil enzymes takes on a vital importance to nutrient mineralization, organic matter decomposition, and plant nutrient cycling (Li et al., 2015b). Soil enzyme activity also serves as a sensitive indicator of soil environmental changes (Tan et al., 2023). In general, it is correlated with soil physical and chemical properties, indicates the direction and intensity of soil biochemical processes, while significantly affecting soil physical and chemical properties, fertility, and biological conditions. Accordingly, it commonly serves as an essential indicator to evaluate the environmental quality of the soil (Guo et al., 2020; Cheng et al., 2022). Previous research suggested that the application of organic fertilizers generally increases soil enzyme activity in comparison with soil where fertilizers are applied, thus indicating higher nutrient cycling capacity. For instance, the addition of pig manure notably increases enzyme activity (Zhang et al., 2019). In contrast, chemical fertilizer application reduces the activity of C and N-associated hydrolases (DeForest et al., 2011). The functional capacity of soil microbial communities is manifested by the activity of enzymes that play a certain role in the nutrient mineralization process (Miao et al., 2019). For example, soil microbes produce enzymes associated with carbon and nitrogen acquisition (e.g., cellulase, urease, leucine aminopeptidase, N-acetyl-β-D-glucosaminidase, and invertase) (Tao et al., 2015; Liu et al., 2017; Zhang et al., 2017). Thus, exploring the effect of partial substitution of organic fertilizer on soil bacterial community and enzyme activity is of great significance.

However, there has been scarce research on the response of soil enzyme activity, bacterial community composition and diversity to partial replacement of chemical fertilizer by organic fertilizer in maize farmland in the mountain red soil area of Yunnan though some studies have suggested that partial substitution of chemical fertilizer with organic manure is capable of improving soil nutrients, bacterial community diversity, and soil enzyme activity. In addition, the results of the effects of fertilization measures on soil bacterial communities remain controversial in different soil types and soil environments in different regions. Accordingly, a hypothesis was proposed in this study, i.e., replacing chemical fertilizer with nitrogen (e.g., base-applied organic manure) will enhance soil properties, microbial properties, and enzyme activities. This study primarily investigated (1) the effects of partial substitution of chemical fertilizer with organic manure on soil physicochemical properties, bacterial diversity and community composition, and enzyme activity, and (2) the correlation between soil properties, soil bacterial communities and enzyme activities.

## 2. Materials and methods

### 2.1. Experimental design and soil sampling

The positioning test was established in May 2016 at the Practice Teaching Experimental Station of Yunnan Agricultural University, Daheqiao, Xundian County, Kunming City, Yunnan Province, China (25°31′07″N, 103°16′41″E), and this test began in May 2018, and the experimental design and treatment are the same every year. This region has a temperate Subtropical monsoon climate, with an altitude of 1860 m, a mean annual precipitation of 900 ~ 1,000 mm, and a mean annual temperature of 15.80°C. The soil is classified as a mountain red soil (mountain red soil is a subclass of red soil) in the Chinese Soil Taxonomy. The cropping system was a continuous monocropped maize (*Zea mays* L.). The main soil properties (0–20 cm depth) are elucidated as follows: pH, 6.84, organic matter, 28.57 g·kg^−1^, total nitrogen, 109.45 mg·kg^−1^, nitrate N, 77.10 mg·kg^−1^, available P, 14.25 mg·kg^−1^, and available K, 129.37 mg·kg^−1^.

The experiment was completely randomized design. This site has 18 plots (6 treatments and three replicates per treatment), with the respective plot of 28 m^2^ (7 m × 4 m). The corn planting distance is 60 cm, the plant spacing is 25 cm, the ditch is sowed, the sowing depth is 4–5 cm, and 280 plants are planted in each community. The six different treatment fertilization methods in this study are elucidated as follows: no fertilization (CK), N, P and potassium (K) inorganic fertilizer (M0), the percentage of manure replacing chemical fertilizer 10%(M10), 20%(M20), 30%(M30), 40%(M40), organic manure application was 1,162 kg·ha^−1^, 2,325 kg·ha^−1^, 3,487 kg·ha^−1^, 4,650 kg·ha^−1^, respectively. The tested organic fertilizer was pig manure. The amount of nutrient inputs (N, P_2_O_5_, and K_2_O) was the same for the five treatments. The inorganic fertilizer (M0): N, P_2_O_5_, and K_2_O (240, 75, and 75 kg·ha^−1^) was employed in the maize, respectively. Table 1 lists the amounts of N, P_2_O, and K_2_O from chemical fertilizer and manure for the respective treatment. Organic fertilizer and phosphorus and potassium fertilizer are used as a one -time strip of base fertilizer under the seeds. The total amount of nitrogen applies is 40% as the base fertilizer, 60% of the total amount of nitrogen are used for additional fertilizer (25% of the total nitrogen). Weeding, watering, and disease -insect worst prevention are performed according to conventional field management.

**Table 1:** Application rate of chemical fertilizer /organic manure for different fertilization treatment (kg·ha^−1^).

Treatment	Chemical fertilizer			Organic fertilizer		
	N application rate	P_2_O_5_ application rate	K_2_O application rate	N content	P_2_O_5_ content	K_2_O content
CK	0	0	0	0	0	0
M0	240	75	75	0	0	0
M10	216	62.46	64.91	24	12.54	10.09
M20	192	49.92	54.82	48	25.08	20.18
M30	168	37.38	44.73	72	37.62	30.27
M40	144	24.84	34.64	96	50.16	40.36

Soil samples were collected from the respective plot at 0–20 cm depth at the uprooting stage of the fifth crop cycle (18 September 2018). Fresh soil samples were immediately transported to the laboratory in an ice-box for in-depth processing. Gravel and residual roots were removed, and the soil samples were sieved (2 mm). Subsequently, a portion of the respective sample was stored at 4°C and then analyzed for enzyme activities and soil microbial biomass (MBC). Another part was stored at −80°C for microbiological Illumina MiSeq sequencing analysis. The rest of each sample was air-dried for chemical analysis.

### 2.2. Measurements of soil chemical properties and microbial biomass carbon

Soil pH was extracted at a ratio of 1:2.5 (soil: water) and determined using pH meter (HANNA, HI2221). Total nitrogen (TN) was determined using semi-trace Kjeldahl method. Soil nitrate nitrogen (NO_3_^−^-N), ammonium nitrogen (NH_4_^+^-N)1 mol L^−1^ KCl leaching, 1 h filtration by shaking, and the filtrate was determined directly using a flow analyzer (Yang and Zhang, 2022). Total soil organic carbon (TOC) was determined using the TOC-VCPH organic carbon analyzer. The supernatant was pumped with a 0.45 μm membrane after the soluble organic carbon (DOC) was extracted with distilled water, and the filtrate was determined directly using the TOC-VCPH organic carbon analyzer (Yu et al., 2019). β-glucosidase (BG) activity was indicated by p-nitrophenol colorimetry, and the sample was incubated at 37°C to synthesize 1 μg of p-nitrophenol (PNP) per gram per hour of dry soil (Dick et al., 1996). Cellulase (CBH) activity was examined by 3,5-dinitrosalicylic acid colorimetry, and soil culture at 37°C was expressed by producing 1 μg of reducing sugar per gram per hour of soil sample (Yang et al., 2014). Invertase (INV) activity was examined through colorimetry using 3,5-dinitrosalicylic acid, and then the sample was cultured at 37°C to synthesize 1 mg of glucose per hour per gram of soil sample (Gong et al., 2019). Urease (URE) activity was incubated at 38°C using the indigophenol blue colorimetric method to yield 1 μg of NH_3_-N per gram per hour of soil sample (Dick et al., 1996). Leucine aminopeptidase (LAP) activity was examined through chromogenic reaction by p-nitroaniline, and then the sample was cultured at 37°C to synthesize 1 μmol of p-nitroaniline per hour per gram of soil sample per day. Soil N-acetyl-β-glucosidase (NAG) was chromogenic by p-nitrophenol chromogenic and then incubated at 37°C to generate 1 μg of p-nitrophenol (PNP) per gram per hour of dry soil (Bell et al., 2013).

### 2.3. Soil DNA extraction, amplification, and sequencing

0.5 g of fresh soil was extracted using the soil DNA isolation kit FastDNAr SPIN (MP Biomedicals, Santa Ana, United States) in accordance with the instructions provided by the manufacturer. DNA mass and concentration were determined using a spectrophotometer (NanoDropTM 2000 (Thermo Fisher Scientific, Waltham, United States)). The V4-V5 hypervariable region of the 16S rRNA gene was selected and then amplified with primers 515F (5’-GTGCCAGCMGCCGCGG-3′) and 907R (5’-CCGTCAATTCMTTTRAGTTT-3′). The PCR reaction was performed in a mixture of 20 μL consisting of 2 μL of 10 × buffer, 2 μL of deoxyribonucleoside triphosphate (dNTPs) (2.5 mmol L^−1^), 0.8 μL (5 μmol L^−1^) per primer, 0.2 μL of conventional Taq polymerase, 0.2 μL of BSA, 10 ng template DNA, and double distilled water. The PCR conditions were 95°C for 3 min, 35 cycles, 95°C for 30 s, 55°C for 30 s, 72°C for 45 s, and 72°C for 10 min. The PCR reaction was repeated 3 times. PCR products were extracted from 2% agarose gels, polymerized at equal concentrations, sequenced with the Illumina MiSeq platform, and high-quality sequences grouped into operational taxons (OTUs) at a 97% sequence similarity level. For the respective representative sequence, the classification information was annotated using the Unite database^1^ based on the Blast algorithm using the QIIME software (Version 1.9.1).^2^ It is noteworthy that total soil DNA extraction, amplification, library construction, sequencing and data analysis were performed by Beijing Novo Technology Co., Ltd., China.

### 2.4. Statistical analysis

The differences in soil properties, soil enzyme activity, soil bacterial α diversity and bacterial community composition between treatments was evaluated using One-way ANOVA (ANOVA), and the possible correlations between bacterial community, bacterial diversity, soil properties and soil enzyme activity was explored through Pearson correlation analysis. ANOVA and Pearson analysis was conducted using SPSS 23.0 statistical software. The complexity of sample species diversity was studied using Alpha diversity based on four indicators (i.e., Shannon, Simpson, Chao1, and ACE). All indicators in the sample were obtained using QIIME (version 1.7.0) and displayed using R software (version 2.15.3). Moreover, the differences in species complexity of the samples were evaluated through Beta diversity analysis, and the beta diversity of weighted and unweighted single plants was determined using QIIME software (Version 1.7.0). The dimensionality of the original variables was reduced through principal component analysis (PCA) using the FactoMineR package and ggplot2 package in the R software (Version 2.15.3) before clustering. Non-metric multidimensional scale (NMDS) maps were used based on the Bray–Curtis dissimilarity to visualize changes in bacterial community structure between different samples. Furthermore, the effects of soil properties and soil microbial communities on soil enzyme activity were investigated through redundancy analysis (RDA).

## 3. Results

### 3.1. Soil physiochemical properties, microbial biomass and maize yield

**Figure 1:**
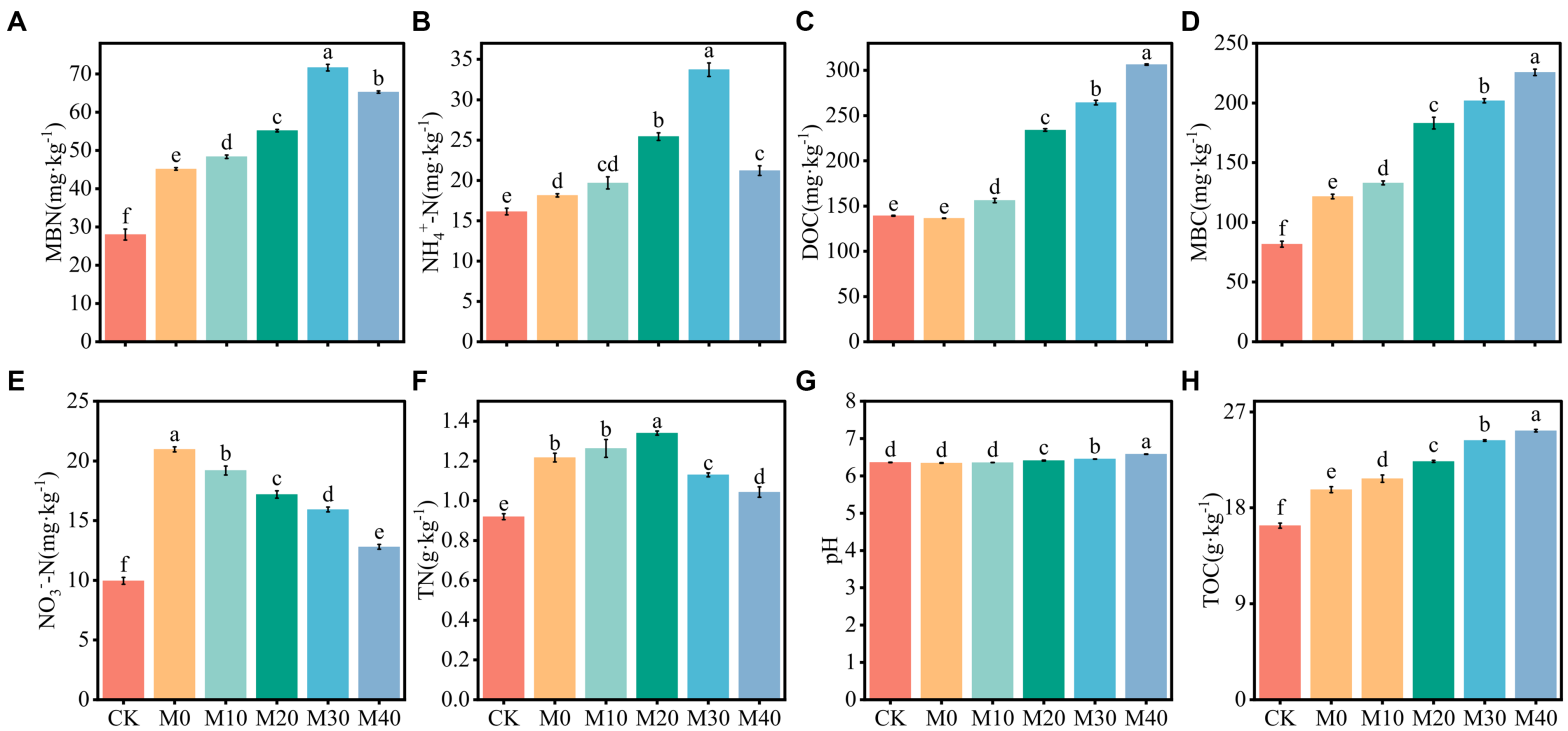
Change characteristics of soil physical and chemical properties and microbial biomass under different fertilization treatments **(A-H)**. Values followed by different letters are notably different (*p* < 0.05) according to Tukey’s multiple comparison test. TN, pH, NH_4_^+^-N, NO_3_^−^-N, TOC, DOC, MBC and MBN represent total nitrogen, soil pH, ammonium nitrogen, nitrate nitrogen, total organic carbon, dissolved organic carbon, microbial biomass carbon, microbial biomass nitrogen, CK, without fertilizer. M0, single inorganic fertilizer. M10, M20, M30 and M40 stand for nitrogen of organic manure replacing 10% chemical nitrogenous fertilizer, nitrogen of organic manure replacing 20% chemical nitrogenous fertilizer, nitrogen of organic manure replacing 30% chemical nitrogenous fertilizer, nitrogen of organic manure replacing 40% chemical nitrogenous fertilizer. The same as below.

Base organic manure substituting chemical fertilizer with equal nitrogen had an effect on soil nutrient content and microbial biomass to varying degrees (Figure 1). In contrast to CK, the MBN and MBC contents of M10, M20, M30 and M40 increased significantly by 72.56–155.66% and 62.72–176%, respectively (Figures 1A,D), the NH_4_^+^-N content increased by 22.05–109.07% (Figure 1B), the NO_3_^−^-N content grew by 28.47–92.76% (Figure 1E), and the TN content increased notably by 13.41–45.65% (Figure 1F), DOC and TOC contents rose obviously by 12.11–120.03% and 27.02–54.56%, respectively (Figures 1C,H). Compared with M0, the MBN and MBC contents of M10, M20, M30 and M40 increased notably by 7.05–58.59% and 9.26–85.32%, the content of NH_4_^+^-N increased by 16.95–85.84%, the NO_3_^−^-N content decreased by 8.46–38.96%, and the DOC and TOC increased by 14.38–124.49% and 5.17–27.98%, respectively. The pH differed significantly between M20, M30 and M40 (*p* < 0.05). In summary, the contents of DOC, TOC and MBC and pH reached the higher values at the organic manure substitution ratio of 40%, the MBN and NH_4_^+^-N content were peaked at the organic manure substitution ratio of 30%, the NO_3_^−^-N content decreased as the organic manure substitution ratio increased, and the TN content reached the higher values at the organic manure substitution ratio of 20%.

**Figure 2:**
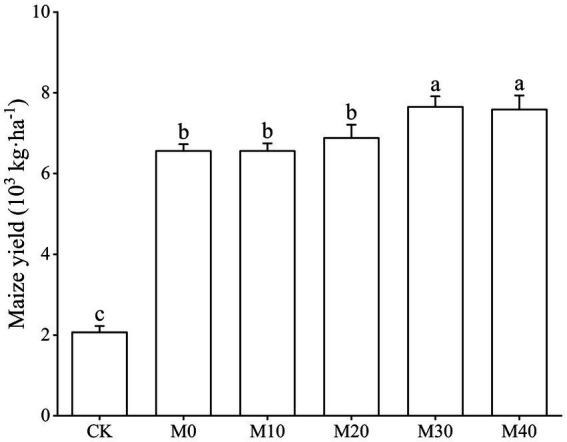
Change characteristics of maize yield under different fertilization treatments.

Base organic manure substituting chemical fertilizer with equal nitrogen had an effect on maize yield to varying degrees (Figure 2). The order of maize yield was M30 > M40 > M20 > M10 > M0 > CK. Compared with M0, M10 and M20 treatments, the maize yield of M30 treatment increased significantly by 16.69, 16.66 and 11.16%, and that of M40 treatment increased significantly by 15.65, 15.62 and 10.17%.

### 3.2. Soil enzyme activities

The activities of CBH, INV, BG, LAP, NAG, and URE were significantly increased by 81.06–302.93% compared with CK, and significantly increased by 23.99–183.16% compared with M0, and the activities of INV, BG, and URE were significantly different among treatments. In brief, the activities of CBH, INV, and BG increased as the proportion of organic fertilizer substitution rose, and their activities reached the maximum at the substitution ratio of 40%, while LAP, NAG, and URE exhibited the highest activities under the substitution treatment of 30%.

### 3.3. α-Diversities of bacterial communities

Partial substitution of chemical fertilizer with organic manure changed the diversity and richness of soil bacteria to varying degrees (Table 2). Compared with CK treatment, the Chao1 and ACE indices of M10, M20 and M30 increased by 49.61–72.8% and 48.27–71.04%, respectively, and the Shannon indices of M10, M20, M30 and M40 increased remarkably by 82.91–116.74%. Compared with M0 treatment, the Chao1 and ACE indices of M10, M20 and M30 were significantly increased by 68.05–94.04% and 66.16–91.68%, respectively, and the size orders of Chao1 and ACE indices were illustrated as M30 > M20 > M10 > M40 > CK > M0. The Shannon index of M10, M20, M30, and M40 treatment increased remarkably by 92.42–128.01% compared with M0 treatment, in which the Shannon index under M20 treatment reached the maximum. The Simpson index did not differ significantly between treatments.

**Table 2:** Variation characteristics of microbial *a*-diversity under different treatments.

Treatments	Chao1	ACE	Shannon	Simpson
CK	554.64 ± 82.73b	556.14 ± 68.84b	2.95 ± 0.13b	0.75 ± 0.06a
M0	493.80 ± 6.68b	496.27 ± 5.48b	2.81 ± 0.36b	0.75 ± 0.14a
M10	829.82 ± 30.13a	824.61 ± 31.41a	5.40 ± 0.42a	0.90 ± 0.04a
M20	922.28 ± 123.23a	908.87 ± 122.44a	6.40 ± 0.53a	0.94 ± 0.03a
M30	958.42 ± 100.83a	951.23 ± 98.40a	5.43 ± 0.57a	0.85 ± 0.04a
M40	585.98 ± 10.99b	596.67 ± 1.82b	5.42 ± 1.43a	0.88 ± 0.06a

Date: Values are mean ± standard deviation (*n* = 3), different letters in the same column represent significant differences at the *p* < 0.05 level.

### 3.4. Soil bacterial community structure and composition

**Figure 3:**
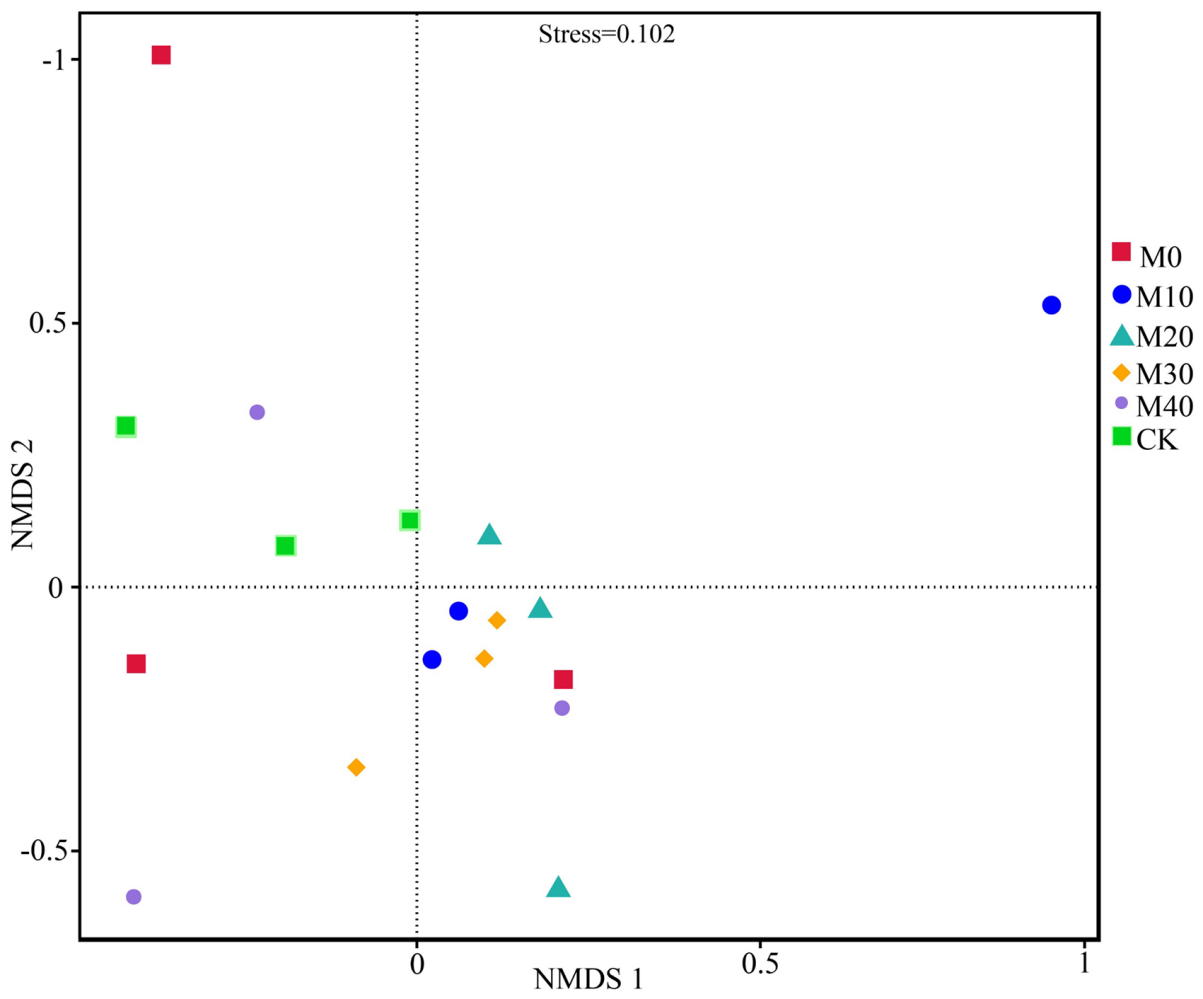
Changes in soil bacterial beta diversity (NMDS) under different fertilization treatments.

**Figure 4:**
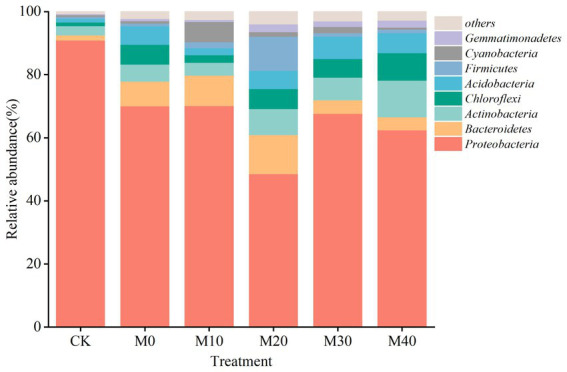
The 16S rRNA gene-based bacterial phylum community compositions in six treatments.

**Figure 5:**
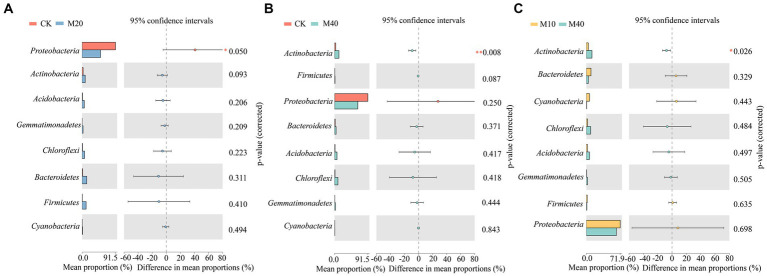
Differences in the relative abundance of bacteria in communities at the phylum levels **(A-C)**. The number of asterisks indicates significant differences between treatments according to a one-way ANOVA. Tukey–Kramer was used as a Post-hoc test and FDR (False Discovery Rate) adjustment (*p* < 0.05): ^*^0.01 < *p* ≤ 0.05; ^**^0.001 < *p* ≤ 0.01; ^***^p ≤ 0.001.

Non-metric multidimensional scale analysis (NMDS) was performed (Figure 3) to compare the bacterial community composition under different fertilization treatments. A significant separation was identified between CK treatment and M10, M20 and M30 (stress = 0.102), whereas no significant difference was reported between M40 and CK treatment, and there was no significant difference between M0 and the respective treatment. Different proportions of substitution of chemical fertilizer with organic manure treatment affected the composition of soil bacterial community to different degrees (Figures 4, 5). The relative abundances of *Proteobacteria* under CK, M0, M10, M20, M30 and M40 treatments were determined as 90.87, 70.04, 70.15, 48.53, 67.64 and 62.46%, respectively, and the abundance of *Proteobacteria* was reduced by different substitutions of chemical fertilizer with organic manure treatments. Compared with CK treatment, the relative abundance of *Proteobacteria* under M20 treatment decreased by 46.6% (Figure 5A), whereas the relative abundance of *Actinobacteria* under M40 treatment increased by 301.53% (*p* < 0.01) (Figure 5B). *Actinobacteria* differed notably between the M10 and M40 treatments (*p* < 0.05) (Figure 5C). *Firmicutes*, *Bacteroidetes*, *Chloroflexi*, *Cyanobacteria*, *Acidobacteria* and *Gemmatimonadetes* did not differ remarkably between treatments.

### 3.5. Relation between soil physicochemical properties and biological properties

**Figure 6:**
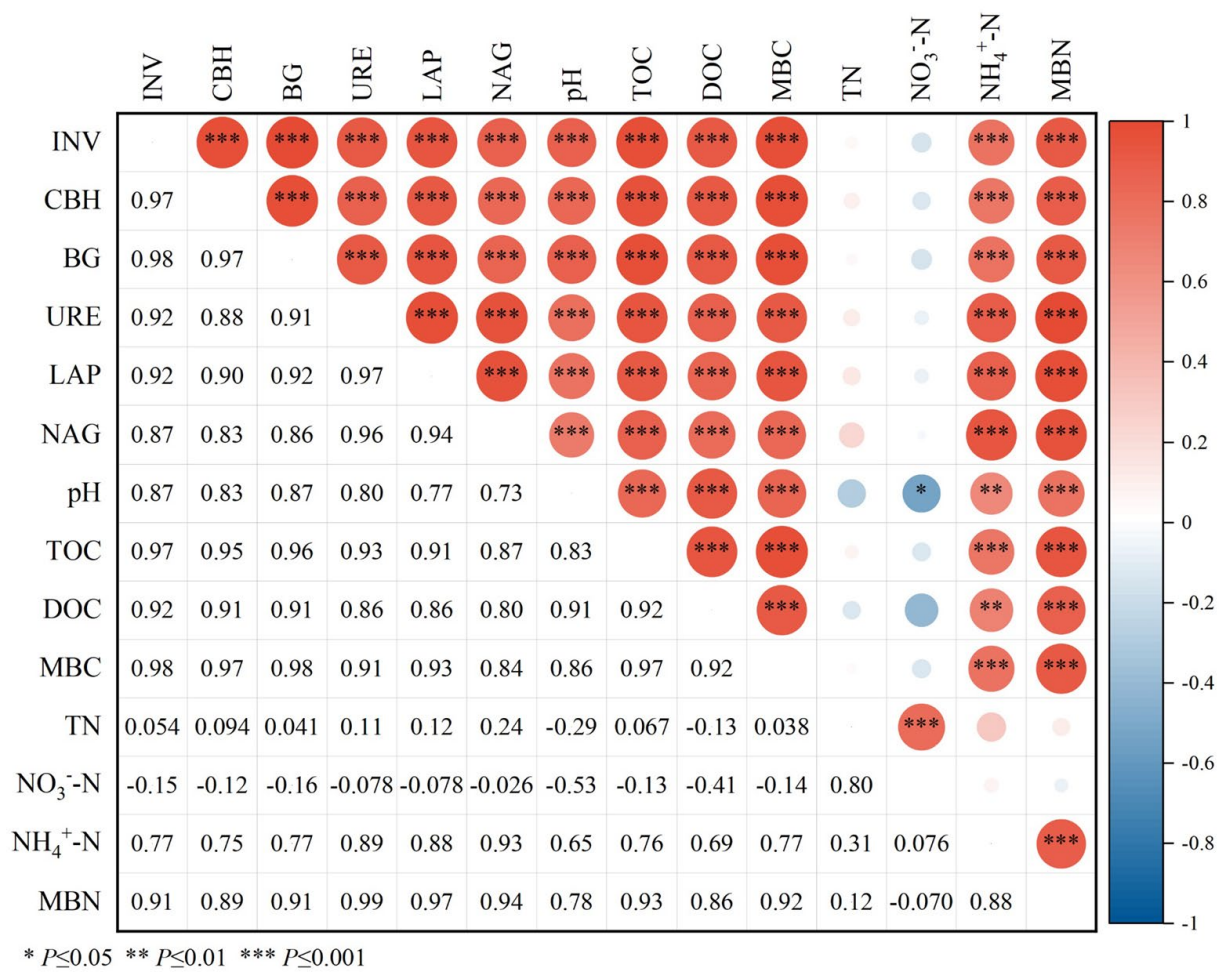
Spearman correlation between soil enzyme activities and physicochemical properties. pH, TN, NH_4_^+^-N, NO_3_^−^-N, TOC, DOC, MBC, MBN, INV, CBH, BG, URE, LAP and NAG represent soil pH, total nitrogen, ammonium nitrogen, nitrate nitrogen, total organic carbon, soluble organic carbon, microbial biomass carbon, microbial biomass nitrogen, Invertase activity, Cellulase activity, BGcosidase activity, Urease activity, Leucine aminopeptidase activity, acetyl--D-glucosaminidas activity.

Spearman was used to analyze the correlation between soil physicochemical properties and soil microbial mass, carbon and nitrogen and enzyme activity (Figure 6). The results showed that the activities of carbon-metabolizing enzymes (INV, CBH and BG) and nitrogen-metabolizing enzymes (URE, LAP and NAG) were positively correlated with pH, TOC, DOC, NH_4_^+^-N, MBC and MBN (*p* ≤ 0.001), and the correlation coefficients were between 0.73–0.98, and the correlation coefficient was not significant between TN and NO_3_^−^-N.

**Figure 7:**
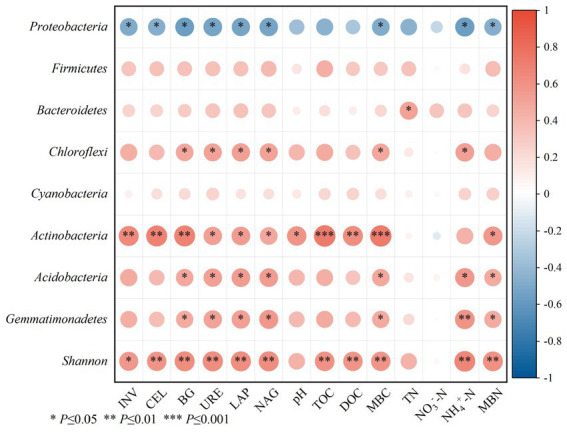
Correlation analysis between bacterial communities at the phylum levels and bacterial *a*-diversity with the soil physicochemical properties and enzyme activity.

**Figure 8:**
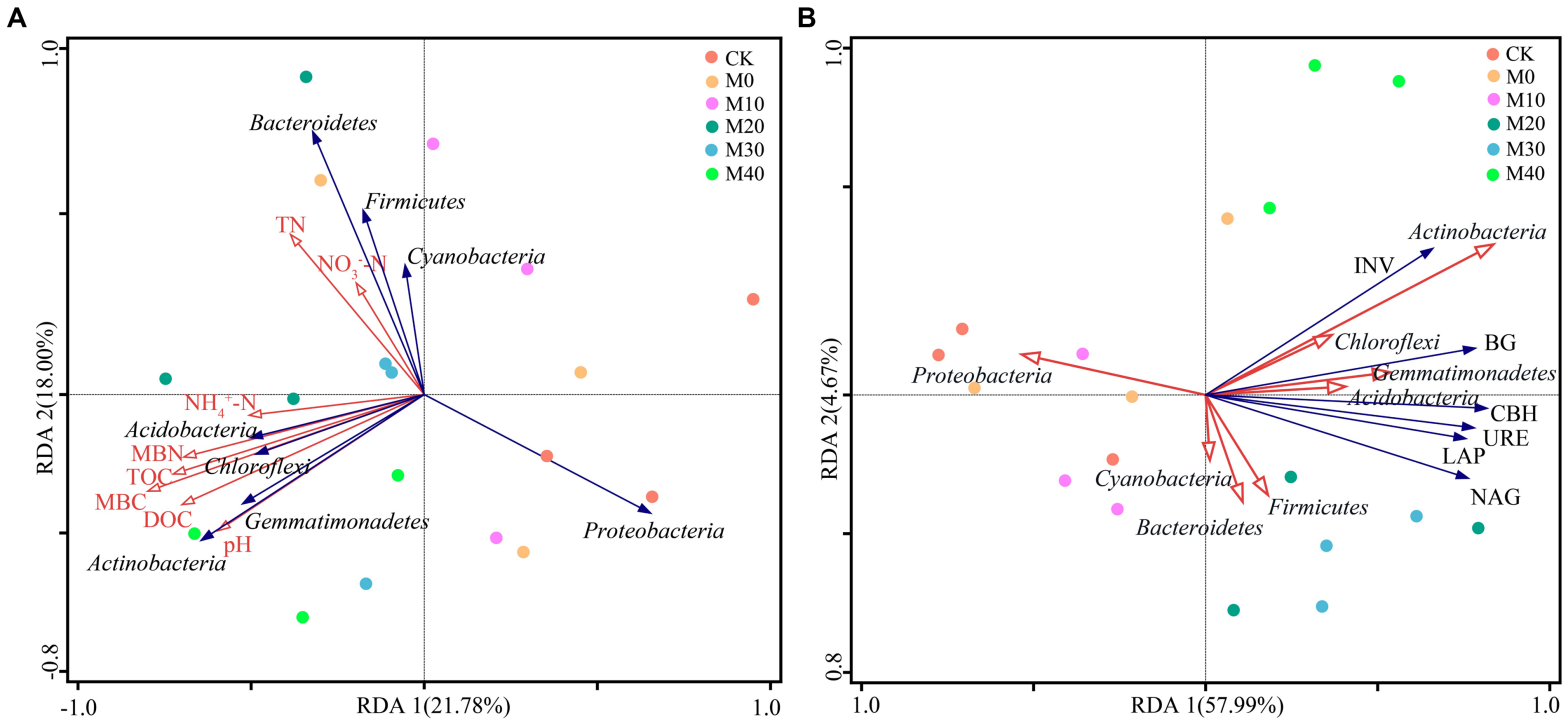
Panel **(A)** presents the redundancy analysis (RDA) between phylum level bacterial communities (blue arrow) and soil physicochemical properties (red arrow) under different fertilization treatments. Panel **(B)** illustrates the redundancy analysis (RDA) between phylum level bacterial community (red arrow) and soil enzyme activity (blue arrow) based on different fertilization treatments.

The effects of soil physicochemical properties, soil enzyme activity and microbial mass carbon and nitrogen on microbial community structure were analyzed using Spearman and RDA (Figures 7, 8). *Proteobacteria* showed a significant negative correlation with INV, CBH, BG, URE, LAP, NAG, MBC, MBN, and NH_4_^+^-N (*p* < 0.05). *Bacteroidetes* had a remarkable positive correlation with TN (*p* < 0.05). Notably, the relative abundance of *Chloroflexi* phylum was positively correlated with BG, URE, LAP, NAG, MBC and NH_4_^+^-N (*p* < 0.05). The relative abundance of *Actinobacteria* phylum showed a notable positive correlation with INV, CBH and BG (*p* < 0.01), with URE, LAP, NAG, pH and MBN (*p* < 0.05), and with TOC, DOC, and MBC (*p* < 0.001). Both *Acidobacteria* and *Gemmatimonadetes* had a remarkable positive correlation with BG, URE, LAP, NAG, MBC, MBN and NH_4_^+^-N (*p* < 0.05), and the Shannon index achieved a significant positive association with INV, CBH, BG, URE, LAP, NAG, MBC, MBN, TOC, DOC, and NH_4_^+^-N (*p* < 0.05) (Figure 7).

The first and second axes accounted for the changes of bacterial communities by 21.78 and 18.00%, respectively (Figure 8A), as revealed by redundancy analysis. In the redundant analysis of soil physicochemical properties and microbial mass, and pH, DOC, TOC, MBN, MBC and NH_4_^+^-N were positively correlated with *Actinobacteria*, *Acidobacteria*, *Gemmatimonadetes* and *Chloroflexi*. TN and NO_3_^−^-N were positively correlated with *Bacteroidetes*, *Firmicutes*, and *Cyanobacteria*, whereas pH, DOC, TOC, MBN, MBC, NH_4_^+^-N, NO_3_^−^-N and TN were negatively correlated with *Proteobacteria*. In the redundant analysis of bacterial community and soil enzyme activity, the first and second axes accounted for the change of bacterial community by 57.99 and 4.67%, respectively (Figure 8B), and INV, CBH, BG, URE, LAP and NAG were positively correlated with *Actinobacteria*, *Gemmatimonadetes*, *Acidobacteria*, *Chloroflexi* and *Firmicutes*, while they were negatively correlated with *Proteobacteria*. MBC (*p* = 0.022) was the main factor for the composition of bacterial community under the treatment of organic fertilizer partial replacement fertilizer, while *Actinobacteria* (*p* = 0.008) was the major factor for enzyme activity.

## 4. Discussion

### 4.1. Effects of partial substitution of chemical fertilizer with manure on soil physicochemical properties and microbial biomass

Soil physicochemical properties are considered an indicator of soil quality; in general, they are defined as the ability to maintain soil environmental quality and improve crop yields (Li et al., 2020; Ren et al., 2021). However, soil microbial biomass plays a certain role in nutrient transformation and cycling in terrestrial ecosystems and serves as a reservoir of available nutrients for plants (Wang et al., 2021). In this study, chemical fertilizer was partially substituted with organic manure, thus leading to the significantly increased soil nutrient content and microbial biomass (Figure 1). To be specific, the DOC, TOC, and MBC contents reached the maximum at 40% organic manure substitution ratio, while MBN and NH_4_^+^-N contents reached the maximum at the organic manure substitution ratio of 30%, consistent with the results obtained by Lazcano et al. (2012). Luan et al. (2020) also suggested that microbial biomass and soil C content increased with the increase of organic manure substitution ratio, probably because organic manure provides sufficient C and nutrient resources, thus facilitating organic carbon sequestration (Muhammad et al., 2020) and microbial growth and reproduction (Mi et al., 2016). Besides, DOC is active soil organic matter that is easily decomposed by soil microbes, and it is critical to improving soil nutrients, microbial biomass and enzyme activity (Li et al., 2018). In addition, the presence of microbes in organic manure may contribute to the increase of soil microbial biomass (Dong et al., 2014). In this study, compared with no fertilization treatment, different proportions of substitution of chemical fertilizer with organic manure treatment notably increased the NO_3_^−^-N content, whereas the NO_3_^−^-N content decreased remarkably with the increase of the proportion of organic manure substitution. The above result is consistent with the results obtained by Yang et al. (2020). In their study, the chemical nitrogen fertilizer was substituted with organic manure to study NO_3_^−^-N in wheat field soil, the reason may be that after the application of organic manure, on the one hand, it increased the input of carbon and increased the soil C:N ratio, resulting in the fixation of N (Hou et al., 2023), on the other hand, organic manure promotes nitrogen uptake by crops (Abbasi and Tahir, 2012), reduced the apparent excess of nitrogen and the accumulation of NO_3_^−^-N, thereby reducing the content of NO_3_^−^-N (Wen et al., 2016). Xu (1996) also indicated that organic manure is capable of alleviating nitrate accumulation and leaching in soil profiles. Hou et al. (2022b) reported that the content of AN, NO_3_^−^-N and MBN in the soil is significantly increased when the substitution rate of organic manure is less than 20% or between 20 and 40%. In this study, however, the partial substitution of chemical fertilizer with organic manure increased the soil nitrogen nutrient content to varying degrees, consistent with the previous research results. In this study, the proportion of organic manure substitution by 20–40% notably increased soil pH, and the pH increased with the increase of organic manure substitution ratio (Figure 1G), consistent with the results of existing research such as (Ding et al., 2016). The possible reason for the above result is that organic acids containing carboxyl and phenolic hydroxyl groups in manure can buffer soil acidity (García-Gil et al., 2004). Furthermore, pig manure is weakly alkaline, which has the potential to neutralize soil acidity, which can remarkably alleviate soil acidification caused by nitrogen, phosphorus, and potassium fertilization (Sun et al., 2015). However, no significant correlation was reported between pH and soil microbial community in this study, not consistent with the results achieved by Yang et al. (2018). The possible reasons for this result are different soil and climate factors.

### 4.2. Effects of partial substitution of chemical fertilizer with organic manure on soil enzyme activities

**Figure 9:**
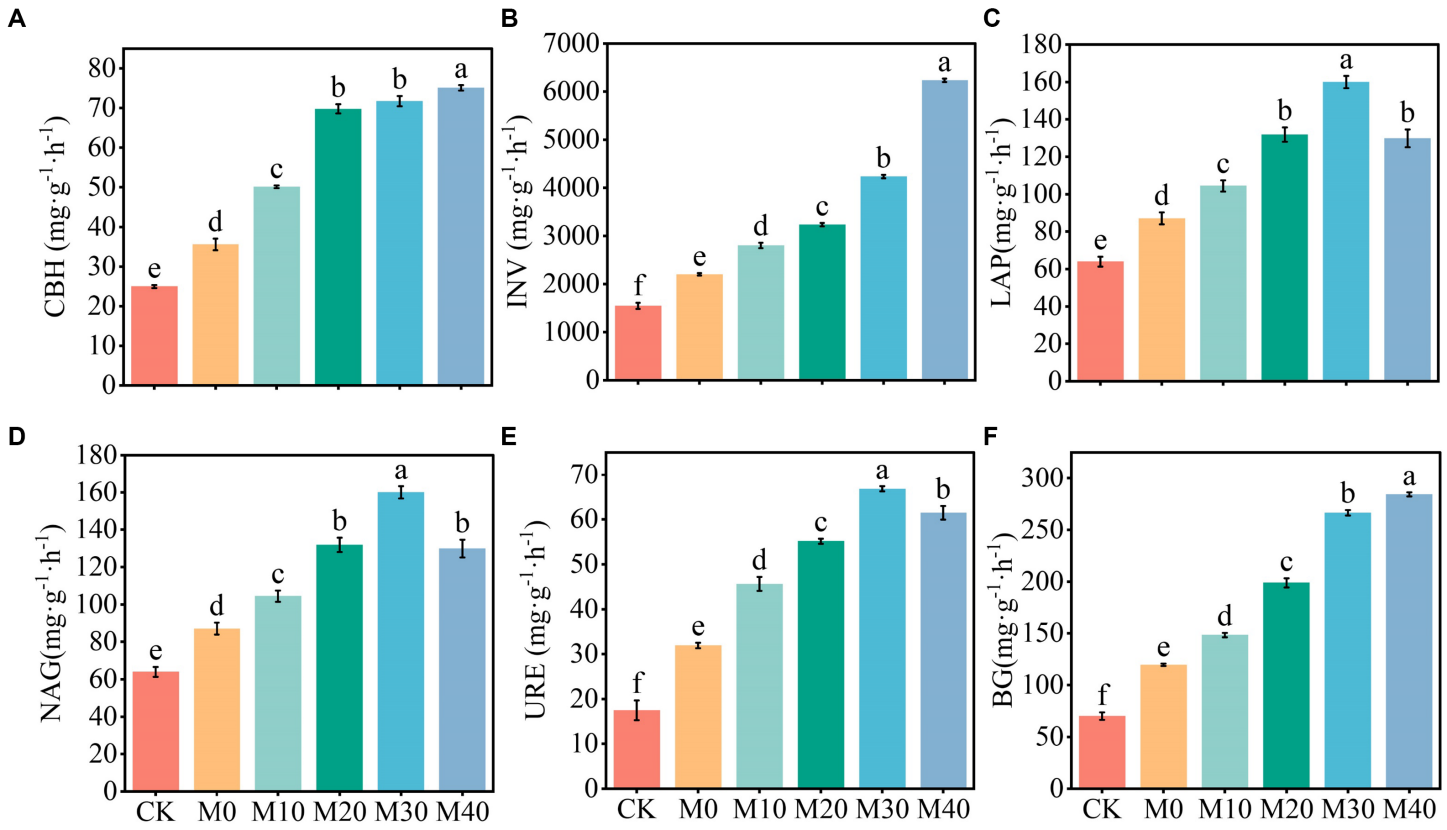
Soil enzymes activity for the respective treatment **(A-F)**. Different letters mean significant difference(*p* < 0.05)between different treatments, INV, CBH, BG, URE, LAP and NAG represent Invertase activity, Cellulase activity, β-glucosidase activity, Urease activity, Leucine aminopeptidase activity, β-1,4-N-acetylglucosaminidase activity, the same as below.

Soil enzymes play an important role in soil nutrient cycling and metabolism and have a direct impact on soil fertility (Hu et al., 2016; Xiao et al., 2018). In this study, carbon-metabolizing enzyme activity (CBH, INV, and BG) increased with the increase of organic fertilizer replacement ratio, and nitrogen-metabolizing enzyme activity (LAP, NAG, and URE) showed the same trend, whereas it was peaked at the substitution ratio of 30% (Figure 9). The major reason for the above result is that compared with single application of chemical fertilizer and CK, organic manure directly or indirectly increased soil humus (organic matter) content through crop growth; thus, the protective sites of soil enzymes, and the carbon and nitrogen sources of soil microorganisms increased, microbial reproduction was facilitated, and the increase of enzyme activity was stimulated (Gu et al., 2009; Li et al., 2022). Second, organic matter, the substrate of soil enzyme, may also directly induce the increase of soil enzyme activity. Although the substitution of chemical fertilizer with organic manure effectively alleviates the carbon limitation in soil, excessive alternative fertilizer may cause increased nitrogen competition (Olander and Vitousek, 2000). As a result, the consumption of considerable nitrogen metabolism enzymes in the soil should meet the soil nitrogen supply, such that the substitution ratio of 40% organic manure is lower than that of 30% soil nitrogen metabolism enzyme activity. Besides, Tao et al. (2015) and Zhang et al. (2020) suggested that partial substitution of chemical fertilizer with organic manure is capable of significantly increasing the activity of INV, URE, and CBH. The study of Ning et al. (2017) achieved similar results, i.e., a high proportion of organic manure supplementation resulted in higher enzyme activity. The possible reason for this result is that the addition of organic manure facilitates the growth of microbes and the increase of organic substrates is correlated with enzyme synthesis, such that the enzyme activity involved in major plant macronutrients is enhanced (Dinesh et al., 2010). In addition, the results showed that CBH, INV, LAP, NAG, URE and BG activities were positively correlated with pH, TOC, DOC, NH_4_^+^-N, MBC and MBN (Figure 6), which was similar to the results of positive correlation between hydrolase activity and TOC, DOC, and TN found in Hojjati and Nourbakhsh (2006). The possible reason for this result is that soil enzymes facilitate macromolecular organic hydrolysis, thus increasing carbon and nitrogen content, and sufficient C and N resources (derived from manure) can alleviate the restriction of soil microbial metabolism on C and N, thus promoting enzyme secretion (Wang et al., 2010). In this study, 10–40% of the organic manure substitution ratio notably improved the soil enzyme activity, and the soil carbon and nitrogen metabolism enzyme activity was basically consistent with the change of soil carbon and nitrogen nutrients, and the substitution of chemical fertilizer with basic organic manure can remarkably enhance the soil biological characteristics in the short term, thus facilitating the transformation and storage of soil nutrients and enhancing soil fertility.

### 4.3. Effects of partial substitution of chemical fertilizer with manure on soil bacterial diversity and community composition

Soil microbes take up an essential part of terrestrial ecosystems, and the diversity and community structure of microbes are not only closely correlated with the effectiveness of biotransformation, but also reflect soil fertility status and plant health, so the changes of microbial diversity and community have been widely concerned by soil scientists (Manasa et al., 2020; Yang and Zhang, 2022). Bacteria are considered a more accurate measure of soil fertility in comparison with fungi because they have much shorter turnaround times than fungi in utilizing carbon substrates (Demoling et al., 2007; Kuzyakov, 2010). Tian et al. (2015) found in short-term (3-year) field trials that the application of organic manure significantly reduced soil bacterial diversity. However, Han et al. (2021) reported that the partial substitution of chemical fertilizer with organic manure notably increases the species richness of bacteria, Chao1 and Shannon index, consistent with the results in this study, i.e., the proportion of organic manure substitution of 10–30% remarkably increased the Chao1, ACE and Shannon index, whereas the bacterial richness did not increase obviously at the proportion of 40% organic manure substitution. The reason for this result is that high mineral fertilizer inputs or excessive application of organic manure may inhibit bacterial growth, thus resulting in a decline in soil bacterial diversity (Cai et al., 2017; Ji et al., 2018), while moderate organic manure substitution ratios provide available C and N sources for soil microbes, thus contributing to the increase of bacterial diversity and richness (Xu et al., 2017). In this study, the single application of chemical fertilizer and 40% substitution ratio inhibited the growth of some bacteria, resulting in lower bacterial diversity. Furthermore, the bacterial *α* diversity index (Shannon) showed a notable positive correlation with enzyme activity (INV, CBH, BG, URE, LAP, NAG), MBC, MBN, TOC, DOC, and NH_4_^+^-N (*p* < 0.05) under different substitutions of chemical fertilizer with organic manure treatment (Figure 7), consistent with the results of the Shannon index of soil bacterial community diversity found in Lu et al. (2022) and showing a significant positive correlation with pH, MBC, and URE. Long-term application of organic manure boosts the development of soil bacterial community diversity, mainly due to the effects on soil chemistry, biological properties and enzyme activities, especially on soil microbial biomass carbon (Li et al., 2015a; Miao et al., 2019). Moreover, as revealed by the above analyses, the improvement of soil physicochemical properties and soil enzyme activities can facilitate the growth and reproduction of soil bacteria.

In this study, the main bacterial phylums detected were *Proteobacteria*, *Bacteroidetes*, *Actinobacteria*, *Chloroflexi*, *Acidobacteria*, which are similar to those found in other agricultural soils applied with organic manure (Wu et al., 2014; Ji et al., 2018). *Proteobacteria* is the dominant gate under each fertilization treatment, which is consistent with most studies (Ji et al., 2021). *Proteobacteria* is the most abundant phylum in soils and exists in environments with high nutrient levels and can be grown and metabolized using unstable C (Trivedi et al., 2013; He et al., 2021). This study found that the abundance of *Proteobacteria* was reduced by different substitutions of chemical fertilizer with organic manure treatments compared with no fertilization treatment, and the organic manure substitution ratio was more significant (*p* < 0.05) (Figure 4). The reason for this phenomenon may be that the application of organic manure causes certain specific bacterial groups to have a “preference” response due to the increase in available organic compounds, consuming large amounts of nutrients and inhibiting the growth of *Proteobacteria* (Lin et al., 2019). However, Yang and Zhang (2022) showed that the soil bacterial community characteristics of citrus orchards under partial replacement of bio-organic fertilizer showed that different organic fertilizer partial fertilizer treatment increased the relative abundance of *Proteobacteria*, which is contrary to the results obtained in this study. This may be partly due to different crop types, organic manure types, soils, and climate types (Xue et al., 2016). In addition, *Actinobacteria* under 40% organic manure substitution treatment was notably improved compared with no fertilization and 10% organic manure substitution treatment (*p* < 0.05) (Figure 5). *Actinobacteria* had a remarkable positive correlation with pH, DOC, TOC, MBN, MBC, NH_4_^+^-N, and enzyme activity (INV, CBH, BG, URE, LAP, and NAG) (Figure 7) since *Actinobacteria* refers to a highly abundant phylum of bacteria in a good nutrient environment that takes on a critical significance to soil carbon and nitrogen cycling (Zhu et al., 2019), which can contribute to the rapid growth of lignin, hemicellulose, protein, cellulose, and other C/N substances (Dai et al., 2018). Moreover, the relative abundances of *Bacteroidetes*, *Actinobacteria*, *Chloroflexi*, *Acidobacteria*, *Firmicutes*, *Cyanobacteria* and *Gemmatimonadetes* at the proportion of 10–40% organic manure substitution were higher than those without fertilization, whereas the relative abundance did not increase or decrease as the organic manure substitution ratio rose (Figure 4). The possible reason for this result is the different degree of response of each bacterial phylum to the proportion of organic manure application. As revealed by previous research, *Bacteroidetes* can degrade complex substances (e.g., starch, cellulose, lignin, and pectin) (Zhu et al., 2019), and nitrogen content may directly or indirectly cause the transfer of dominant flora members, especially *Bacteroidetes* (Fierer et al., 2012). In this study, the relative abundance of *Bacteroidetes* based on different treatments was higher than that of CK, consistent with the results of existing research (Yang and Zhang, 2022). The possible reason for this result is *Bacteroidetes* preference for eutrophication environments, where the application of organic manure increases soil nutrients while boosting *Bacteroidetes* use of carbon sources (Liang et al., 2018). *Acidobacteria and Gemmatimonadete* showed a significant positive correlation with enzyme activity (BG, URE, LAP, NAG), MBC, MBN, and NH_4_^+^-N, similar to the results of the study (Zhang et al., 2014), a class of oligotrophic bacteria that are less distributed in nutrient-rich agricultural soils (Lopez-Lozano et al., 2013). Studies have shown that soil properties are closely correlated with soil microbial community structure and can affect soil microbes to varying degrees (Xun et al., 2015), and soil microbial mass carbon and enzyme activity are important indicators of soil biological activity (Song et al., 2022). Ren et al. (2021) studied the effect of partial substitution of chemical fertilizer with organic manure on soil microbial community and soil enzyme activities. Their result suggested that soil bacterial community significantly affected soil enzyme activities. Through the RDA analysis of soil properties, this study also confirmed that soil enzyme activity and soil bacterial community phylum level, MBC (*p* = 0.022) is the main factor for the composition of bacterial community under the treatment of partial substitution of chemical fertilizer with organic manure, while *Actinobacteria* (*p* = 0.008) is the main factor for soil enzyme activity (Figure 8), consistent with the previous results.

## 5. Conclusion

In summary, this study shows that the alternative fertilizer of organic manure has significantly improved the nature of soil physical and chemical, maize yield, soil bacterial community diversity and enzyme activity. Based on these four aspects, 30% of organic manure substitution ratio is better. Among them, the maize yield of 30 and 40% of organic manure substitution treatments was significantly higher than that of CK and 0–20% of organic manure substitution treatments, and the yield of 30% of organic manure substitution treatment was the highest, reaching 7652.89 kg·ha^−1^. Compared with fertilization treatment, the relative abundance of *Proteobacteria* under M20 is significantly reduced by 46.60%. INV, CBH, BG, URE, LAP, and NAG activity are very positive as pH, TOC, DOC, NH_4_^+^-N, MBC, MBN. Shannon Index and enzyme activity (INV, CBH, BG, URE, LAP, and NAG), MBC, MBN, TOC, DOC, and NH_4_^+^-N are significantly positive. MBC is the main factor affecting bacterial community composition under the partial substitution of chemical fertilizer with organic manure, while *Actinobacteria* is the main factor affecting enzyme activity. To sum up, the appropriate proportion of organic manure substitution is conducive to improving soil properties, providing a good living environment for bacterial communities, thereby increasing soil enzyme activity, improving soil micro-ecological environment, and achieving sustainable agricultural development.

## Data availability statement

The original contributions presented in the study are included in the article/Supplementary material, further inquiries can be directed to the corresponding authors.

## Author contributions

Experimental work and draft manuscript were completed by ZeL and YZ. Experimental ideas are provided by YL, CL, ML, XS, ZhL, JZ, and MF. All authors contributed to the article and approved the submitted version.

## Funding

This study was funded by the National Key Research and Development Program of China (2022YFD1901500 and 2022YFD1901502).

## Conflict of interest

The authors declare that the research was conducted in the absence of any commercial or financial relationships that could be construed as a potential conflict of interest.

## Publisher’s note

All claims expressed in this article are solely those of the authors and do not necessarily represent those of their affiliated organizations, or those of the publisher, the editors and the reviewers. Any product that may be evaluated in this article, or claim that may be made by its manufacturer, is not guaranteed or endorsed by the publisher.

## Footnotes

^1^ https://unite.ut.ee/

^2^ http://qiime.org/scripts/assign_taxonomy.html
